# Advances in Bone-on-a-Chips for In Vitro Modeling of Bone Physiology and Pathology

**DOI:** 10.3390/biomedicines14030710

**Published:** 2026-03-19

**Authors:** Xiuyun Cheng, Mingxia Lu, Ming Ma, Shumin Zhou, Jun Xu, Yuhao Li, Hongxu Lu

**Affiliations:** 1 School of Materials and Chemistry, University of Shanghai for Science and Technology, Shanghai 200093, China; 18721816977@163.com (X.C.); yhli@usst.edu.cn (Y.L.); 2 Biomaterials and Tissue Engineering Research Center, Shanghai Institute of Ceramics, Chinese Academy of Sciences, 1295 Dingxi Road, Shanghai 200050, China; 3 State Key Laboratory of High Performance Ceramics, Shanghai Institute of Ceramics, Chinese Academy of Sciences, 1295 Dingxi Road, Shanghai 200050, China; mma@mail.sic.ac.cn; 4 Institute of Microsurgery on Extremities, Shanghai Jiaotong University Affiliated Sixth People’s Hospital, Shanghai 200233, China; zhoushumin_zw@126.com; 5 Department of Orthopaedic Surgery, Shanghai Jiaotong University Affiliated Sixth People’s Hospital, Shanghai 200233, China; junxu19781214@163.com

**Keywords:** bone-on-a-chip, microfluidics, bone microenvironment, bone organoids

## Abstract

Bone is a dynamic and multifunctional tissue that provides mechanical support, regulates mineral homeostasis, supports hematopoiesis, and relies on complex interactions among multiple cell types. The increasing incidence of bone-related diseases, such as osteoporosis, osteoarthritis, fracture non-union, and bone cancer, highlights the need for in vitro models that better reflect human bone physiology. Bone-on-a-chip technology, developed through advances in microfluidics, biomaterials, and tissue engineering, offers a promising approach to recreate key features of the bone microenvironment in vitro. By incorporating bone-mimicking materials, relevant bone cells, vascular components, fluid perfusion, and mechanical stimulation, these platforms allow more realistic investigation of bone remodeling, regeneration, disease mechanisms, and drug responses. In parallel, bone organoids and their integration with microfluidic chips have further expanded the capabilities of in vitro bone models by enabling the formation of self-organized, human-relevant bone tissues with increased cellular complexity. This review summarizes recent progress in bone-on-a-chip systems, including models for osteogenesis and bone regeneration, vascularized bone, bone marrow and hematopoietic niches, cancer bone metastasis, and mechanobiological studies. Key design principles, materials, cellular components, and applications in disease modeling, drug screening, toxicity assessment, and personalized medicine are discussed. Current challenges and future directions are also discussed to support the continued development of more physiologically relevant in vitro bone models.

## 1. Introduction

The skeletal system is a vital component of human structure and function. Bones play a crucial role in providing support and attachment to body muscles, protecting vital organs such as bone marrow and the brain, and acting as metabolic organs with major calcium and phosphate reserves [1]. With the exacerbation of global population aging and the increase in the obese population, the number of patients with bone diseases such as osteoporosis, osteoarthritis, osteosarcoma, and bone metastasis is continuously increasing, and the incidence of bone diseases is also on the rise. The current treatment options for these diseases are limited, and the treatment process is painful, with the repair effects often falling short of the ideal state. Many patients are unable to achieve full recovery and may even face more serious health issues as well as significant economic burdens [2,3]. Consequently, research on bone diseases has been receiving growing attention. Currently, bone disease research mainly relies on cell experiments and animal experiments, which have significant limitations, including large differences from human natural bones, high costs, and long experimental cycles [4]. To address these issues, researchers have explored new approaches.

**Figure 1:**
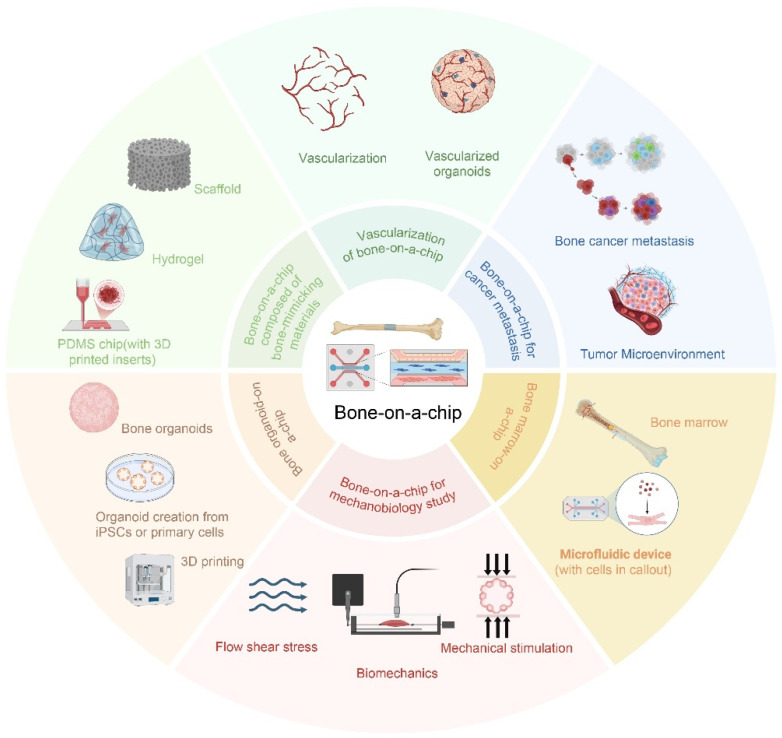
The construction and application of bone-on-a-chip for biomedical applications. These include bone-on-a-chip for regeneration, vascularized bone-on-a-chip, bone marrow-on-a-chip, bone-on-a-chip for metastasis, bone-on-a-chip for mechanobiological studies, and bone organoids.

Microfluidic technology is a technique that precisely controls fluids within micrometer-scale spaces. It utilizes microfabrication techniques to design the structures of microchannels, as well as to accurately regulate the driving methods of fluids and the properties of fluids themselves. This enables the mixing, separation, and reaction of fluids at the microscale [5]. By integrating microfluidic chip technology to construct bone-on-a-chip, a better simulation of the dynamic microenvironment of bone tissue can be achieved, offering a new pathway for the study of mechanisms of bone remodeling, disease models, bone tissue engineering, and the effects of drugs on the skeleton [6] (Figure 1).

To achieve this goal, researchers can use materials and microfluidic technology to simulate the hard and dense nature of cortical bone and the porous and spongy characteristics of trabecular bone. At the same time, the construction process of the bone-on-a-chip will also involve the main types of bone cells, including osteoblasts, osteocytes, and osteoclasts, to simulate their roles in the physiological and pathological processes of bone. In addition, the simulation of the bone matrix is a key part of the bone-on-a-chip design. The bone matrix contains organic and inorganic components, with the organic part mainly being Type I collagen and the inorganic part being hydroxyapatite crystals. In the bone-on-a-chip, biological materials can be used to simulate these components, thereby providing a growth environment for cells that is similar to natural bone. Microfluidic technology can also be used in the bone-on-a-chip to simulate mechanical factors in the skeleton, such as fluid shear forces and pressure, which have a significant impact on the behavior of bone cells and the physiological processes of the skeleton. In this way, the bone-on-a-chip can not only provide an accurate simulation of the microenvironment of bone cells but also provide an experimental platform for studying the response of bones to mechanical stimulation.

## 2. Bone and Bone-Related Diseases

### 2.1. Bone Biology

The skeleton is primarily composed of the hard and dense cortical bone on the outer side and the porous and spongy trabecular bone on the inside [7]. Microscopically, it is primarily composed of bone matrix and cells, mainly including three types: osteoblasts, osteocytes, and osteoclasts.

Bone matrix is mainly composed of inorganic components and a variety of organic components; the organic part is primarily Type I collagen and other proteins, while the inorganic part is mainly hydroxyapatite crystals [8]. The inorganic substances provide mechanical rigidity and load-bearing strength to the skeleton, while the organic matrix offers elasticity and flexibility [7]. These components of the skeleton work in concert to ensure the health and function of the bones.

Bone cells are primarily composed of osteoblasts, osteocytes, and osteoclasts, which work together in a highly coordinated manner to maintain the balance of the skeletal system. These cells form a multicellular unit that precisely regulates the continuous cycle of bone resorption and bone formation. This is a key mechanism to ensure bone balance and skeletal health [9]. Osteoblasts are responsible for synthesizing new bone matrix, regulating the mineralization of the matrix, and forming a network of osteocytes. Osteocytes represent terminally differentiated osteoblasts and function within syncytial networks to support bone structure and metabolism. Osteoclasts are the only cells that are known to be capable of resorbing bone, originating from mononuclear macrophages. Osteoclasts can dissolve the minerals and organic components of the bone matrix by secreting enzymes and acidic substances, thereby resorbing bone tissue. They also participate in the bone remodeling process and regulate the renewal and repair of bone tissue [7,10].

Bone remodeling is crucial for repairing microdamage, maintaining the mechanical strength of the skeleton, and regulating the body’s calcium and phosphorus balance [7]. The remodeling process is mainly composed of three consecutive phases: resorption, reversal, and formation. During the resorption phase, osteoclasts are activated and attach to old bone tissue, and they digest the bone matrix by secreting acidic substances and enzymes, leading to bone absorption. After osteoclasts have completed absorption, the reversal phase begins, during which mononuclear cells appear on the bone surface. During the formation phase, osteoblasts are attracted to the absorbed bone surface and begin to produce and secrete ECM, which is then mineralized to form new bone tissue. After the bone matrix matures, osteoblasts transform into osteocytes, which connect to each other through cellular processes, forming a network of osteocytes [11]. The three stages of bone remodeling are continuous and strictly regulated to ensure the health and integrity of the skeleton.

### 2.2. Bone-Related Diseases

An imbalance in the coordination process of several osteocytes will destroy bone homeostasis and lead to severe bone diseases [1], including osteoporosis, fracture healing, osteoarthritis, primary/metastatic bone tumors, etc.

Osteoporosis is currently one of the most common bone diseases. It is estimated that more than 200 million patients have osteoporosis, and 50% of women and 20% of men over the age of 50 will suffer an osteoporosis-related fracture. Fractures may lead to long-term disability and reduced quality of life, increasing the risk of death [12,13]. Osteoporosis is caused by an imbalance in the process of bone remodeling when estrogen levels are reduced, and aging leads to increased bone resorption and decreased bone formation [1,14,15]. Osteoporosis not only causes fractures but also promotes bone metastasis [16].

Fracture is also a common type of bone disease. After the fracture, the inflammatory reaction occurs first, followed by soft tissue callus and hard callus formation. Finally, the repair is completed through the bone remodeling stage. In this process, osteoblasts form new bone, while osteoclasts are involved in bone resorption. These two work together and coordinate to maintain the balance of bone tissue [17].

Osteoarthritis is a chronic and progressive degenerative disease of the whole joint. It causes severe joint stiffness and pain, and may eventually lead to disability, which seriously affects patients’ quality of life. Osteoarthritis is not only irreversible; there is no cure [18]. In the early stages of osteoarthritis, abnormal mechanical loads on the joint cause changes in the microstructure of the subchondral bone, leading to excessive activation of osteoclast bone resorption. This is a key pathological feature of osteoarthritis [19].

Primary bone cancers make up less than 1% of all cancer cases, while bone metastases from other cancers are quite common, particularly in adults [20]. The term “primary bone cancer” describes tumors that originate specifically in bone tissue from bone cells. In contrast, some cancers, such as breast cancer, can spread to the bones. Primary bone cancers can be mostly classified into four categories: (i) osteosarcoma (OS), which typically affects the leg and arm bones of children and young adults; (ii) Ewing sarcoma, which usually develops in the pelvis, legs, or arms of similar age groups; (iii) chondrosarcoma, where cancerous cells form cartilage; and (iv) malignant fibrous histiocytoma. Bone metastasis is a common late-stage manifestation of some cancers, including breast, lung, kidney, and thyroid cancers [20]. The condition of cancer patients with bone metastases is serious. Bone metastases can lead to pain, spinal cord compression, fractures, bone marrow aplasia, and hypercalcemia [21].

At present, bone tissue-related diseases pose a significant threat to patients’ health and quality of life, so it is essential to study these diseases in depth. It is hoped that bone tissue disease-related models can be established in vitro to better understand these diseases’ pathogenesis and provide a powerful experimental platform for drug screening and disease treatment.

### 2.3. Challenges in Developing In Vitro Bone Models

Most studies on bone development and diseases have used conventional in vitro 2D cell culture systems or in vivo animal experiments. A 2D cell culture provides a relatively simple experimental environment but cannot fully simulate cells’ 3D structure and morphology in the in vivo environment. It limits the intercellular communication and expression of tissue-specific functions and may not accurately predict the response of drugs in vivo. In sum, the simple 2D monolayer culture model cannot simulate bone [4,22]. Animal models play a significant role in disease research, but there are still genetic differences between animals and humans. The development and maintenance costs of animal models are high, and the experimental cycle is long, limiting their application in drug screening and research. Animal models are also subject to ethical restrictions [23,24]. The replication of some skeletal diseases remains a significant challenge.

## 3. Microfluidic Technology and Organ-on-a-Chip

Microfluidics [25] is a technology for precisely manipulating fluids at the micro- and nanoscale. It has the advantages of miniaturization, automation, and integration, can minimize the time and cost of biological analysis, and can improve reproducibility [5]. An organ-on-a-chip is an in vitro cell culture device based on microfluidic technology that integrates multiple components into a single device, enabling researchers to simulate and reproduce organs in vivo realistically [26,27]. Generally, these chips allow living cells to grow in continuously perfused micron-scale chambers to mimic the physiological functions of tissues and organs. The goal is not to build a complete organ but to reproduce tissue and organ-level function [26], which is able to replicate the physiological responses of single or multiple tissues [27]. The three key features of organ-on-a-chips include: providing a three-dimensional structure for tissues, co-culturing multiple cell types to achieve more physiologically balanced tissue functions, and simulating biomechanical forces, such as simulating the breathing process of lung tissue, the beating of the heart, or the hemodynamic shear forces of vascular tissue [28].

Compared with traditional cell culture and animal models, organ-on-a-chip can reconstruct the complex physiological environment at the microscale and realistically reflect the response of tissues or organs to drugs [29]. Moreover, organ-on-a-chip technology allows precise control and regulation of the mechanical stimulation to which cells are subjected and the application of organ-specific dynamic microenvironments to tissues in vitro, such as fluid shear stress, cyclic strain, mechanical compression, etc. [30]. Organ-on-a-chip technology offers an alternative to traditional animal models, helps reduce the usage of animals in experiments, and may overcome the limitations of animal models in drug development and disease research. Similarly, organ-on-a-chip combines microfluidic technology to simulate tissues and organs at a smaller scale, enabling high-throughput drug screening [31]. In addition, organ-on-a-chip technology can be constructed using a patient’s cells and tissues, enabling personalized medicine which will speed up drug development, reduce costs, and improve success rates.

A variety of organs on chips have been developed, such as lung [32], heart [33], intestine [34], liver [35], kidney [36], brain [37], and multi-organ-on-chips [38], and These chips have been widely investigated in drug screening [39], disease modeling building [40], toxicity evaluation [41], personalized medicine [42], etc. For example, a heart-on-a-chip, which culturing rat cardiomyocytes and containing 16 interfinger electrodes (IDEs) has been designed. The contraction state of cardiomyocytes could be detected using high-speed impedance detection technology. Then, the typical antiarrhythmic drug, verapamil, and the anticancer drug, doxorubicin, were tested using the chip, and the results showed changes in cell activity after drug treatment, indicating that the cardiac effects and toxicity of the drugs can be accurately reflected [39].

Also, the organ-on-a-chip model is promising in disease modeling. For instance, Wang et al. established a liver organoid chip derived from human induced pluripotent stem cells (hiPSCs). This chip has a two-layer structure: the bottom layer is a microcolumn array that supports cell aggregation and the formation of liver organoids. The top layer allows for cell injection and media provision. By exposing liver organoids with free fatty acid (FFA) supplement, it was observed that after FFA induction, liver organoids on the chip showed lipid droplet formation, abnormal triglyceride accumulation, increased markers related to lipid metabolism, and expression of markers of inflammation and fibrosis, which reflected the emergence of pathological features associated with steatohepatitis and approved human non-alcoholic fatty liver disease (NAFLD) was modeled [40].

In the application of toxicity evaluation of organ-on-a-chip, for example, Muhammad et al. [41] developed a multi-sensor lung cancer-on-chip, utilizing a glass-based microfluidic chip with embedded indium tin oxide electrodes for transepithelial electrical resistance impedance sensing, along with an optical pH sensor and a custom 3D-printed digital microscope for visual monitoring, all for the purpose of real-time drug toxicity assessment. Lung cancer cells (NCI-H1437) were cultured on a collagen-coated bottom glass in the ITO region. Then, the toxicity of doxorubicin and docetaxel at different concentrations was evaluated using TEER impedance sensors. In personalized medicine, for example, Hyunho Kim et al. [42] developed a 3D microfluidic co-culture chip that grows three types of cells, including tumor cells from patients with non-small cell lung cancer, immortalized human microvascular endothelial cells, and primary human astrocytes, to simulate the brain tumor microenvironment. It was applied to investigate the relationship between brain metastatic non-small cell lung cancer (NSCLC) cells and the brain tumor microenvironment (bTME), to reflect the response of injected representative targeted drugs across multiple patient-derived tumor cells, and to enable personalized medicine design.

Currently, based on the structural and physiological characteristics of bone tissue, researchers have designed various bone-on-a-chip systems, including models for bone regeneration, vascularized bone, bone marrow, cancer bone metastasis, and mechanobiological studies.

The microfluidic design of bone-on-a-chip requires precise control of channel geometric parameters and fluid dynamic environments. Typical chips employ parallel dual-channel or triple-channel configurations with heights of 200–500 μm and widths of 250–1000 μm, utilizing micropillar arrays (50–100 μm in diameter) to achieve functional separation between cell culture regions and fluidic channels. Hydrodynamic parameter design is grounded in physiological shear stress (0.5–2 Pa) as the core criterion, with flow rates typically set at 2–50 μL/min calculated according to the formula τ = 6μQ/(wh^2^) to generate appropriate fluid shear stress [43,44,45]. Studies have demonstrated that intermittent flow patterns more effectively promote osteogenic differentiation compared to continuous flow. Computational fluid dynamics simulations can predict internal velocity distributions within chips, optimizing channel designs to avoid high shear stress regions (>2 Pa) that may cause cellular damage. PDMS is widely employed due to its optical transparency and moldability, with precise control of channel height ensuring the accuracy of shear stress prediction.

## 4. Development of Bone-on-a-Chip

### 4.1. Bone-on-a-Chip Composed of Bone-Mimicking Materials

Bone-on-a-chip for osteogenic or bone regeneration usually combines a microfluidic chip, biomaterials, and cells to reproduce the bone microenvironment and dynamic processes. Hydroxyapatite (HA), a major inorganic component of bone, is added to the microfluidic chip to assist in building the microenvironment due to its similarity to human bone minerals [46]. Shi et al. [47] manufactured hydroxyapatite (HA) substrates with microchannels using Stereolithography (SLA) technology and assembled the HA-PDMS chip by sealing the HA substrate between a thin layer of polydimethylsiloxane (PDMS). The HA substrates were designed with various structures, including Y-shaped, T-shaped, and Christmas tree-shaped, to simulate complex biological environments.

Compared with pure PDMS chips, the introduction of HA was found to enhance proliferation and osteogenic differentiation of the human fetal osteoblast cell line (hFOB) on the HA-PDMS chips. Subsequently, a Christmas tree structure HA-PDMS chip was chosen to investigate the toxicity of doxorubicin. The chip features a Christmas tree-like branched structure for creating drug concentration gradients. In this design, drugs are introduced through one inlet while a drug-free medium is injected into another, allowing precise flow control to establish varying drug concentrations across different chip regions, which confirms the chip’s application in drug screening [47].

Bone-on-a-chip can be combined with biomimetic bone scaffolds. Native bone tissue exhibits a precisely quantified hierarchical pore architecture. In cortical bone, Haversian canals measure 20–100 µm in diameter and are interconnected via Volkmann’s canals, while canaliculi are submicron-sized (<1 µm), constituting the microscale network for osteocyte nutrient exchange. Cancellous bone demonstrates significant regional heterogeneity in pore diameter: 10–50 µm in cortical regions and up to 300–600 µm in trabecular regions, with actual values varying depending on anatomical location, age, and pathological status. This hierarchical pore size distribution (spanning from submicron to hundred-micron scales) provides a quantitative blueprint for biomimetic scaffold design. Consequently, scaffold pore sizes should be functionally partitioned according to the biological requirements of distinct bone regeneration stages. Small pores (20–100 µm) facilitate cell seeding and early attachment; medium pores (100–200 µm) support cell migration and growth factor delivery; large pores (200–400 µm) are specifically designed for vascularization. Interconnected pores exceeding 400 µm compromise scaffold mechanical integrity without conferring additional biological benefits and are therefore not recommended as routine design parameters [48].

**Figure 2:**
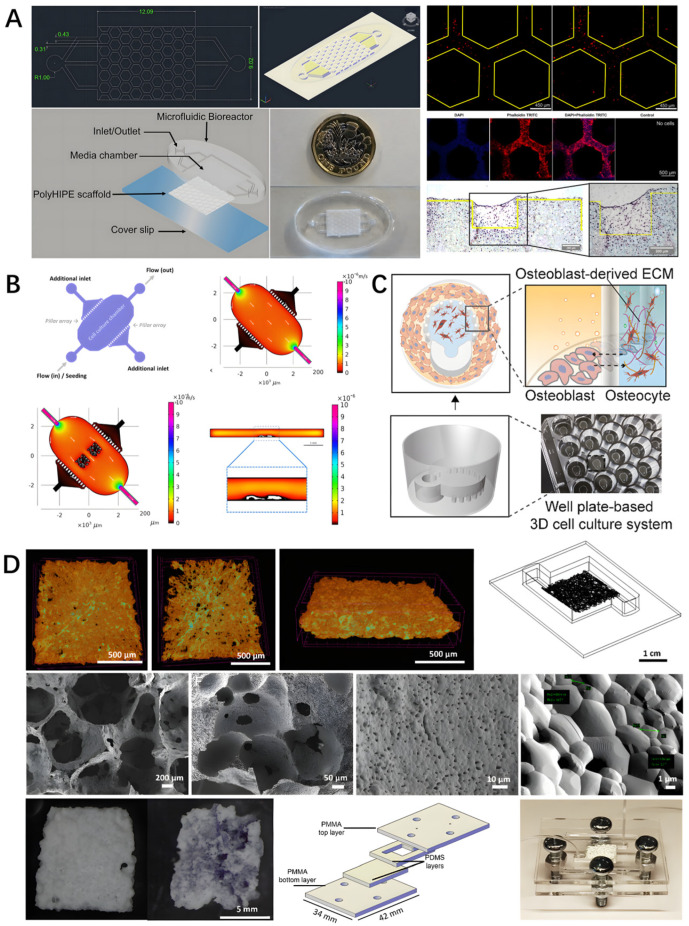
Bone-on-a-chip composed of bone-mimicking materials. (**A**) The design of bone-on-a-chip integrating polyHIPE scaffolds within a PDMS bioreactor, with hexagonal micropillar arrays providing 3D cell culture environment and fluid shear stress; cellular images show osteoblast distribution within the scaffold under intermittent flow conditions (flow rate 2–10 μL/min, shear stress 0.1–0.5 Pa). Reprinted with permission from Ref. [43]. (**B**) The organ-on-a-chip model for bone regeneration featuring a main chamber with inlet/outlet ports and lateral channels separated by micropillars; two-photon polymerization-printed 3D bone structures coated with calcium phosphate simulate trabecular bone macro-pore morphology; computational fluid dynamics simulation shows velocity distribution (0.5–2 mm/s) within the chamber; MSCs cultured for 21 days demonstrate high viability and collagen-rich ECM deposition. Reprinted with permission from Ref. [50]. (**C**) High-throughput biomimetic bone-on-a-chip platform in 24-well plate format with two horizontally aligned compartments; OB-dECM hydrogel provides 3D microenvironment for IDG-SW3 osteocyte and MC3T3-E1 osteoblast co-culture, with immunofluorescence images showing osteocyte marker (DMP1) and osteoblast marker (osteocalcin) expression after 14 days. Reprinted with permission from Ref. [54]. (**D**) β-TCP scaffold-based bone-on-a-chip co-cultured with primary osteoblasts and osteoclast precursors; dynamic culture for 21 days shows dense ECM formation; in vivo implantation into C57BL/6 mice for 8 weeks demonstrates bone formation (blue staining) and remodeling activity (TRAP-positive osteoclasts). Reprinted with permission from Ref. [52].

PolyHIPEs are porous emulsion templates of polymer material synthesized in a high internal phase emulsion [49], which can also be used as a scaffold material in bone-on-a-chips. Bahmaee et al. developed a microfluidic chip to simulate mechanical stimulation in a dynamic environment by leveraging the porous properties of polyHIPEs. The chip consists of a PDMS bioreactor and an embedded polyHIPE scaffold, The 5–30 μm pore size of the polyHIPE scaffold precisely matches the size of osteoblasts, optimizing the interaction between cells and the matrix, which provides a 3D environment and fluid shear stress via repeated hexagonal columns, as shown in Figure 2A. The effects of different flow rates and flow patterns on cell metabolic activity, osteogenic differentiation, and mineralized matrix deposition were investigated. The results showed that cells responded positively to both chemical and mechanical stimulation of osteogenesis. Unlike static and continuous flow, intermittent flow curves with rest periods strongly promoted differentiation and matrix formation. Hydrodynamic simulations showed that the device’s flow rate and shear stress distribution were consistent with physiological conditions in bone tissue [43].

It is also possible to simulate the bone tissue environment by coating the 3D bone structure model with a layer of calcium phosphate, a bone mineral. Calcium phosphate is a substance similar to the inorganic components found in bone tissue, which can provide cells with chemical and physical signals similar to those of natural bone. Researchers first used two-photon polymerization technology to print a 3D bone model in a polymer, Simulate the macroscopic pore morphology of cancellous bone. Then, they coated this 3D model with a layer of calcium phosphate that is similar to bone minerals. Finally, they integrated this 3D bone model into a microfluidic device that is suitable for cell culture. To introduce the chemical properties of the bone, the 3D bone model is then placed into the microfluidic device, where the microfluidic chip is connected by a main chamber with an inlet and an outlet for the flow of the medium and two lateral channels, separated by a series of columns, for the introduction of the CaP solution and cell inoculation, as shown in Figure 2B. Human mesenchymal stromal cells (MSCs) cultured in the platform for up to 21 days showed high viability and collagen-rich bone ECM generation [50].

Beta-tricalcium phosphate (β-TCP) is also commonly used as the main material for bone scaffolds. It is a ceramic biomaterial with good biocompatibility and osteoinductive properties, which can promote new bone formation and the process of bone remodeling [51]. Osteoblasts and osteoclasts were co-cultured using a bioceramic scaffold based on β-TCP. Erbay et al. [52] developed a bone-on-a-chip consisting of a highly porous β-TCP-based scaffold co-cultured with primary osteoblasts and osteoclast precursors, the β-TCP scaffold achieves a multi-level structural simulation of the bone trabecular to bone canal system through a combination of 500–50 μm large pores and 10–1 μm micro pores, as shown in Figure 2D. The cells were cultured dynamically in an organ-on-a-chip for up to 21 days, and a dense ECM was observed. The cultured constructs were implanted ectopically into C57BL/6 mice for 8 weeks, and histological and tartrate-resistant acid phosphatase analysis was performed to evaluate bone formation and remodeling. The results showed good cell proliferation and bone tissue formation of the cell scaffold co-culture in the organ-on-a-chip. The results of in vivo implantation experiments show that the engineered bone tissue chip model can promote bone formation and absorption processes.

The construction of the mechanical microenvironment for bone-on-a-chip requires the mechanical properties of native bone as a reference benchmark. Cortical bone exhibits a Young’s modulus of 15–20 GPa and hardness of approximately 4–6 GPa, whereas trabecular bone demonstrates lower Young’s modulus (0.1–2 GPa) and hardness (0.1–0.5 GPa) attributed to its porous architecture [53]. Existing bone-on-a-chip studies have employed different biomimetic material systems to achieve mechanical property matching with specific bone tissues. Shi et al. selected hydroxyapatite (HA) ceramic as the microfluidic chip substrate; its high stiffness (elastic modulus of approximately 120–130 GPa) matches the mechanical properties of the bone mineral phase [47]. Bahmaee et al. employed polyHIPE scaffolds; this study did not explicitly report the specific Young’s modulus and hardness values of the scaffolds, but rather simulated the hierarchical characteristics of native bone tissue by constructing multiscale porosity [43]. Galván-Chacón et al. [50] simulated bone mechanical properties through a “soft substrate-hard coating” composite structure: using IP-Dip polymer printed by two-photon polymerization as the substrate (2.1 ± 0.3 GPa) with an HA ceramic coating (120–130 GPa) on the surface. Meanwhile, the authors noted that there remains a certain gap between the mechanical properties of the current scaffold and native bone. Erbay et al. [52] employed porous β-TCP scaffolds, utilizing a strategy combining materials with controllable porous structures to simulate native trabecular bone; the reported Young’s modulus was 140.38 ± 9.22 MPa (approximately 0.14 GPa) and compressive strength was 1.72 ± 0.11 MPa, with mechanical properties matching those of native trabecular bone.

Biomimetic microenvironments can be reproduced not only from bone-like materials such as HA, β-TCP, or PolyHIPEs scaffolds, but also from acellular matrices of bone cells, fibrin, or collagen gels. Paek et al. developed a high-throughput bionic bone-on-a-chip platform. As shown in Figure 2C, by setting two horizontally aligned compartments in a 24-well plate, mouse bone cells (IDG SW3) were placed in a three-dimensional gel unit constructed from the osteoblast-derived ECM (OB-dECM) and co-cultured with osteoblasts (MC3T3-E1). Thus, the structural unit of bone is simulated. The combination of natural and bioactive ingredients obtained from OB-dECM and the co-culture of the two osteoblasts synergistically enhance osteogenic functions, such as osteoblast differentiation and maturation [54].

Moreover, a fibrin gel can be used to provide a bone microenvironment. Lee et al. [16] developed a bone-on-a-chip by setting up two channels and designing five triangular pillars to divide the chip into blood vessel and bone microenvironment channels. Fibrin gel provided a bone microenvironment for the co-culture of osteoblasts, osteoclasts, and osteocytes. The ratio of bone cells was adjusted to simulate bone tissue in normal, osteopenia, and osteoporosis conditions, and the use of breast cancer cells to simulate bone metastasis under these conditions showed that bone metastasis was most active under osteoporosis. The results suggest that bone metastasis in osteoporosis is promoted due to increased vascular permeability. The activity of bone cells and the significant increase in osteoclasts may affect the integrity of blood vessels, thereby promoting bone metastasis.

### 4.2. Vascularization of Bone-on-a-Chip

Like other organs, bone also has a stable, persistent, mature vascular network. During bone tissue development, the vascular network plays a critical role in supplying nutrients and oxygen. Concurrently, the interplay between vascular endothelial cells, such as human umbilical vein endothelial cells (HUVECs), and osteogenic progenitor cells, such as MSCs, facilitates the differentiation and maturation of these osteogenic precursors. This cellular crosstalk is essential for bone formation and repair. Additionally, the vascular network serves as a template guiding osteoblasts in the deposition of minerals along vascular structures, thereby promoting the orderly generation of new bone tissue [55]. Jusoh et al. [56] developed a vascularized bone-on-a-chip with four parallel channels. Microcolumns separated the channels, which were filled with endothelial cells, fibrin, and HA nanocrystal scaffolds to simulate the bone tissue matrix with a highly porous, interconnected structure, culture medium, and fibroblasts to induce blood vessel sprouting, separately. Thus, the relationship between different concentrations of HA and angiogenesis was observed, demonstrating that HA could enhance angiogenesis.

Building upon these vascularization strategies, Mainardi et al. developed a modular dual-compartment organ-on-a-chip platform to reconstruct vascularized osteochondral tissues via side-by-side culture of human articular chondrocytes (hACs) and bone marrow-derived mesenchymal stromal cells (bmMSCs). The study demonstrated that co-culture of bmMSCs with human umbilical vein endothelial cells (HUVECs) at a precisely tuned 3:2 ratio facilitates the formation of functional lumenized vascular networks surrounded by α-SMA-expressing pericytes. Under homeostasis, the cartilage layer maintains barrier function by preventing vascular invasion; IL-1β (1 ng/mL) disrupts this barrier, recapitulating early osteoarthritis. This modular platform enables real-time imaging and precise cellular control, offering a robust tool for osteoarthritis research and drug development [57].

### 4.3. Bone-on-a-Chip for Cancer Metastasis

**Figure 3:**
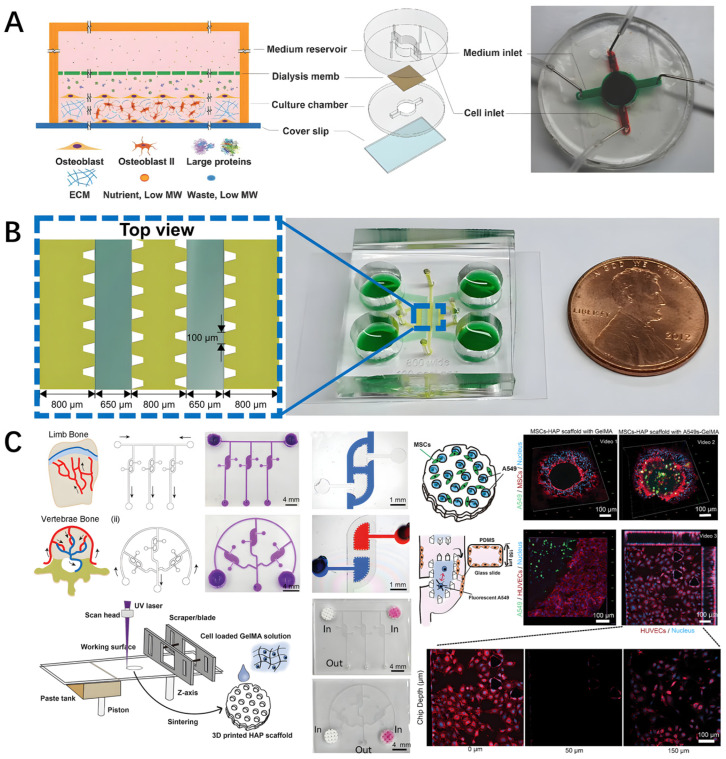
Bone-on-a-chip for cancer metastasis. (**A**) A spontaneous three-dimensional bone-on-a-chip for studying breast cancer bone metastasis, featuring two compartments for osteoblast tissue growth and medium reservoir; mineralized osteoblastic tissue spontaneously forms thick calcium nodules (red staining) within 30 days; subsequent co-culture with MDA-MB-231 breast cancer cells demonstrates rapid tumor cell invasion into mineralized matrix forming micrometastases (green fluorescent cells) with invasive protrusions extending into distant stroma; time-lapse imaging reveals metastatic progression over 72 h. Reprinted with permission from Ref. [59]. (**B**) A three-dimensional microfluidic bone tumor microenvironment composed of hydroxyapatite/fibrin composite; five parallel channels separated by 100 μm gaps with micropillars within channels prevent leakage and trap hydrogels; HA/fibrin composite (HA concentration 0–20%) in the middle channel cultures tumor cells (SW620, MKN74) while lateral channels supply medium; results demonstrate HA inhibits tumor cell migration and proliferation in a dose-dependent manner; co-culture with fibroblasts and endothelial cells reveals reduced angiogenesis at high HA concentrations. Reprinted with permission from Ref. [62]. (**C**) A bone-on-a-chip mimicking the pre-metastatic niche, integrating 3D-printed HA scaffolds (simulating bone matrix), H-type HUVEC channels (modeling vasculature), and gelatin methacryloyl hydrogel infusion chambers; MSCs on scaffolds form dormant niches where tumor cells exhibit reduced invasiveness; osteoclast-tumor cell co-culture in the “vicious circle” niche promotes tumor reactivation and invasion; circulating tumor medium bridges primary tumor and bone metastasis compartments. Reprinted with permission from Ref. [63].

Bone is the preferred site for cancer metastasis because the bone microenvironment supports several growth factors that promote cancer cell metastasis [58]. The mechanism of cancer cell-to-bone metastasis was generally studied by co-culturing cancer cells in a bone-on-chip. Zheng et al. developed a bone-on-a-chip that naturally forms mineralized bone tissue. The chip contained two compartments for osteoblast tissue growth and a medium reservoir, as shown in Figure 3A. It allowed cell growth for up to a month, and thick mineralized osteoblastic tissue formed in vitro. After that, the metastatic human breast cancer cells were co-cultured with osteoblasts in this chip, and the cancer cells rapidly invaded the apical layer of mature, mineralized osteoblasts, forming micrometastases. The invading foot prostrates into the distant stroma, thus observing the pathological features of metastasis [59].

S. Bersini et al. [60] designed a microfluidic chip that was also used to study the specificity of breast cancer metastasis to bone. The microfluidic chip design uses three channels combined with four hydrogel channels. Human osteogenic differentiated bone marrow-derived MSCs were injected into four independent gel channels, embedded in the collagen matrix. They formed hydrogels to simulate the bone microenvironment. After three days, the matrix glue was introduced into the intermediate channel, and the endothelial cells were introduced into the intermediate channel. Three days later, the cancer cells were inoculated again, and their migration to the bone cell area was observed. The results showed that the breast cancer cell receptor CXCR2 and the bone-secreted chemokine CXCL5 play a major role in the exosmotic process, affecting exosmotic rate and travel distance. Finally, it was observed that extravasated cancer cells could proliferate and form micrometastases within the osteoblastic microenvironment, thus demonstrating the power of the bone-specific model [61].

J. Ahn et al. [62] reported a chip to investigate the relationship between the HA, incorporated ECM, and tumor microenvironment. The microfluidic chips consisted of five parallel channels separated by a 100 µm gap, with microcolumns within the channels to prevent leakage and trap hydrogels, as shown in Figure 3B. A 3D bone-simulated complex composed of HA and fibrin was used to culture tumor cells in the middle channel to simulate the bone tumor microenvironment, with the medium channel on both sides. The effects of varying HA concentrations on the activity, proliferation, morphology, and migration of tumor cells (SW620 and MKN74) were studied. Tumor cell migration was significantly reduced, indicating that HA inhibited migration. Fibroblasts could also be cultured through fibrin gel in the lateral two channels, and the tumor microenvironment was simulated by co-culturing tumor cells and fibroblasts. It is also possible to introduce endothelial cell co-cultures and find that intercellular signaling in the tumor microenvironment leads to reduced angiogenesis at high HA concentrations.

The three stages of the pre-metastasis niche can be mimicked via a microfluidic chip. The tumor cells metastasize from the primary tumor to the bone, and a dormant niche will occur. The perivascular niche is a key factor in the process of bone tumor metastasis. The dormant reactivation process of metastatic tumor cells is the “vicious circle” niche. Ji et al. [63] developed a microfluidic platform consisting of a 3D-printed scaffold, a PDMS chip with H-type channels seeded with HUVEC, and a gelatin methacryloyl (GelMA) hydrogel infusion chamber loaded with cells, as shown in Figure 3C. MSCs were seeded on 3D-printed HA scaffolds in the chip to recapitulate resting niches. HUVEC seed channels with an H-shaped design were used to model the vasculature and perivascular niche. For the “vicious circle” niche, osteoclasts and tumor cells encapsulated in a photo-crosslinked GelMA hydrogel were injected into the chip chamber, and their interactions were investigated. Circulating tumor medium (CM) was used as a circulating medium in the channel to bridge the primary tumor and bone metastases. The interaction between tumor cells and the bone microenvironment has been explored. In 3D-printed bone tumor scaffolds, MSCs form dormant niches for tumor cells. Tumor cells showed less invasiveness when co-cultured with MSCs. In the chip co-culture of osteoclasts and tumor cells, tumor cell invasiveness was promoted, leading to increased invasive processes.

### 4.4. Bone Marrow-on-a-Chip

Bone marrow is a complex organ that plays a crucial role in hematopoiesis, maintaining immune system balance, and regulating tumor metastasis [64]. The hematopoietic niche is a complex microenvironment composed of diverse cell populations, secreted factors, and ECM. Based on its location and functional characteristics within the bone marrow, the hematopoietic niche is divided into the endosteal and the perivascular niches. These niches regulate the self-renewal of hematopoietic stem cells (HSCs) and the differentiation of HSC into hematopoietic progenitor cells (HPCs), thereby maintaining the dynamic balance of blood production [65]. Replicating the bone marrow and hematopoietic niche is important for intraosseous studies.

Collins and Ingber et al. [66] utilized tissue engineering to create bone in mice, then implanted it into microfluidic chips to form bone marrow chips for later cell culture. They first used a PDMS device with a central cylindrical cavity, with openings at both ends, filled with type I collagen gel containing osteoinductive decalcified bone powder and bone morphogenetic proteins (BMP2 and BMP4), and implanted it into mice to form new bone containing bone marrow. The structural and compositional similarity of engineered bone marrow (eBM) to natural bone was confirmed by histological analysis, micro-computed tomography (µCT), and energy-dispersive X-ray spectroscopy (EDS). The eBM was then implanted into the microfluidic device, and medium perfusion maintained its function. The eBM has been shown to sustain hematopoietic function in vitro culture and to mimic responses to radiotoxic and radioprotective drugs. As a result of the study, cells harvested from eBM 8 weeks after implantation exhibited a completely normal distribution of HSCS, hematopoietic progenitors, and differentiated blood cells from all lineages. They contained bone marrow with a hematopoietic cell composition nearly identical to that of native bone marrow. The presence of key cellular and molecular components of the hematopoietic niche suggests that the cellular content of eBM closely resembles the natural bone environment.

The bone marrow chip can also replicate multiple human bone marrow niches, including endosteal, central bone marrow, and perivascular niches. Roy et al. [64] designed a human multi-niche microvascularized bone marrow chip in a 96-well plate format. Each plate can be set with eight independent bone marrow devices, each with five channels: a central gel channel, two media channels, and two external gel channels. The five channels are bonded to the bottomless 96-well plate. The wells of the plates were used as media storage tank box channels. The chip was seeded with mesenchymal stem cells in the central channel, and after 21 days of osteogenic differentiation, a bone-like inner bone layer formed, and the cytokines and ECM characteristics of the endosteal niche were expressed. Human vascular endothelial cells and MSCs were seeded in a fibrin-collagen hydrogel, with human vascular endothelial cells and MSCs seeded above them, resulting in an interconnected 3D microvascular network mimicking the central bone marrow and perivascular niches. Thus, human multi-niche microvascularized bone marrow was simulated.

Bone marrow toxicity testing of anticancer drugs was carried out using a bone marrow chip. J. Cairns et al. [67] designed a dual-organ microfluidic chip using TissUse (Humimic Chip 2), consisting of two circuits: a 3D ceramic scaffold (hydroxyapatite) in one compartment and a media reservoir in the other. The pore size and structure of the 3D ceramic scaffold (hydroxyapatite) can mimic human cancellous bone, providing a porous environment for co-culture of CD34+ human stem and progenitor cells on MSC-seeded scaffolds and for media circulation through microfluidic channels to simulate the microenvironment. After co-culture for 7 days, the cells were treated with olaparib or carboplatin, and the collected cells were analyzed by flow cytometry. Olaparib-induced red blood cell toxicity and carboplatin-induced red blood cell and megakaryocyte toxicity indicated that the system could be used to detect the lineage-specific toxicity of drugs to cells.

D. Glaser and his team [68] have developed a vascularized human bone marrow niche chip that successfully mimics the vascularized niches of human bone marrow and can highly replicate a variety of physiological functions of bone marrow. The microfluidic chip consists of two hexagonal chambers connected by three symmetrical bidirectional ports. The port design, based on the capillary burst valve concept, effectively prevents hydrogel leakage into adjacent chambers during loading. Additionally, the chip features an independent bottom chamber that allows loading a third cell type at a later stage of experimentation, providing greater flexibility for multicellular co-culture. To construct vascular networks within each niche, the researchers mixed endothelial cells (ECs) with either osteoblasts (hFOBs) or MSCs in a fibrin hydrogel. They injected the mixtures into the left and right hexagonal chambers, respectively, to create three-dimensional tissues. This design not only successfully mimics the vascularized niches of bone marrow but also replicates the expression of ECM proteins and other key biomarkers found in native bone marrow. Moreover, the chip is capable of studying the interaction between cancer cells and vascularized bone marrow by introducing cancer cells into the third chamber.

### 4.5. Bone-on-a-Chip for Mechanobiology Study

Bones are constantly subjected to mechanical stimuli, and bone remodeling results from osteocytes responding to these stimuli. In vivo, one major mechanical stimulus to osteocytes is fluid shear forces generated by interstitial fluid flowing through the lacunae and tubular networks [69]. Fluid flow provides cells with oxygen, nutrients, waste removal, and mechanical stimulation. Microfluidic chips can provide different levels of fluid shear stress.

Signal transduction between osteocytes and osteoclasts was studied by applying mechanical stimulation with different levels of fluid shear stress. K. Middleton et al. [70] designed a microfluidic co-culture platform. Through three adjacent cell culture channels, a high-resistance side channel was set up between them, and a stable 2 Pa flow rate was applied to one channel that was mechanically stimulated (fOCY), that is, fluid shear stress greater than 0.5 Pa. The fluid shear stresses of the middle channel (OCL) and the other channel, non-mechanically stimulated (nOCY), are 0.28 Pa and 0.07 Pa, less than 0.5 Pa, and are fairly free from mechanical stimulation. Bone cells (MLO-Y4) were cultured in fOCY and nOCY channels, and RAW264.7 osteoclasts were cultured in OCL channels. The results showed that RANKL can promote the differentiation and maturation of osteoclast precursors when used in a RANKL culture medium. The subsequent study investigated changes in the gradients of cytokines (such as RANKL, OPG, and PGE-2) produced simultaneously by osteoclasts in the middle channel and by osteocytes in channels with mechanical stimulation (fOCY) and without mechanical stimulation (nOCY). It was found that the density of osteoclast precursors increases significantly within 200 microns of the nOCY channel, suggesting that mechanical stimulation reduces the RANKL/OPG ratio in osteocytes (MLO-Y4), inhibits osteoclast differentiation and activity, and inhibits osteocyte apoptosis. Reduces signals released by apoptotic osteocytes that promote osteoblast migration and differentiation.

**Figure 4:**
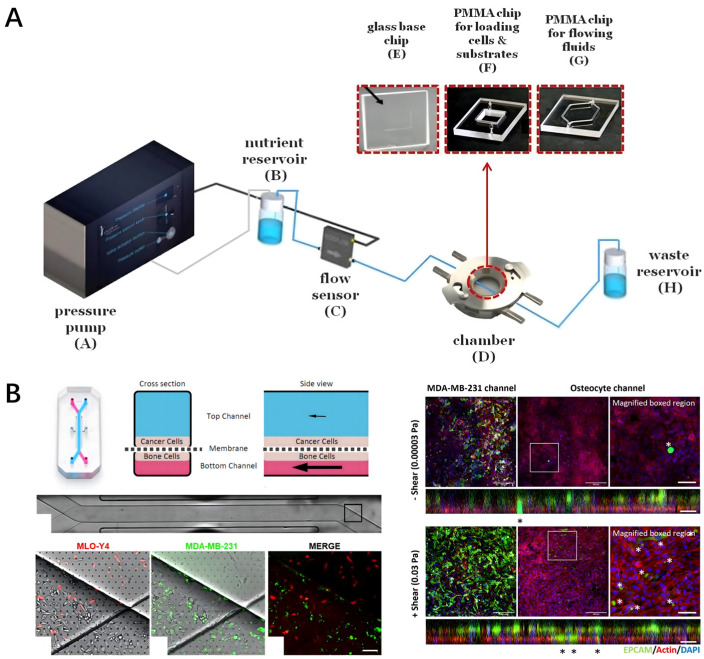
Bone-on-a-chip for mechanobiology study. (**A**) A microfluidic system with precise flow control capability consisting of a pressure pump, nutrient reservoir, flow sensor, microfluidic chamber, and waste reservoir; osteoblasts cultured on gelatin or fibrin gel substrates under static conditions and flow rates of 30 and 50 μL/min (generating shear stress of 0.1–0.5 Pa); phase-contrast images show elongated cell morphology under flow conditions; ALP activity and mineralization assays confirm that mechanical stimulation enhances osteogenic differentiation. Reprinted with permission from Ref. [44]. (**B**) A microfluidic organ-on-a-chip consisting of flexible PDMS elastomers containing two parallel microchannels (height 200 μm, width 500 μm) separated by a porous PDMS membrane (pore size 5–10 μm); the bottom channel cultures MLO-Y4 osteocytes while the top channel cultures breast cancer (MDA-MB-231, MCF-7) or prostate cancer (PC-3, LNCaP) cells; fluid shear stress (0.5–2 Pa) is applied through medium perfusion; results demonstrate that mechanical stimulation increases cancer cell invasiveness and can reverse osteocyte-mediated growth inhibition.The dashed line represents the porous membrane separating the osteocyte and cancer cell culture channels; the arrows indicate the direction of applied fluid shear stress or cell migration. The asterisks denote statistical significance (* *p* < 0.05), indicating that the data in this group is significantly different compared to the control group. scale bar: 20 μm. Reprinted with permission from Ref. [45].

E. Babaliari et al. [44] designed a microfluidic device that can precisely control fluid flow, consisting of a pressure pump connected to a nutrient reservoir, a flow sensor connected to control flow precisely, a chamber containing a microfluidic device, and a waste fluid reservoir, as shown in Figure 4A. Osteoblasts were cultured on gelatin substrate or fibrin gel under static conditions and flow rates of 30 and 50 µL/min to observe the morphology of osteoblasts. The results showed that fluid flow promoted the proliferation and osteogenic differentiation of preosteoblasts.

Breast and prostate cancer tend to metastasize to bone tissue, and the effects of mechanical stimulation and bone cells on the behavior of breast and prostate cancer cells can also be studied. S. Verbruggen et al. [45] utilized a microfluidic organ-on-a-chip model that consists of flexible PDMS elastomers with two parallel microchannels separated by a porous PDMS membrane, one for the cancer cell channel and another for the bone cell channel, respectively, as shown in Figure 4B. Both channels were coated with collagen, and bone cells were cultured in the bottom channel and cancer cells in the top channel. To mimic the microenvironment of bone cells, MLO-Y4 bone cells and breast cancer (MDA-MB-231 and MCF-7) and prostate cancer (PC-3 and LNCaP) cell lines were treated with medium, and mechanical stimulation with fluid shear stress was applied. The results showed that mechanical stimulation can effectively regulate the development of breast and prostate cancer, as well as the osteoblast-cancer cell interaction. They indicated that osteoblast-cell signaling usually inhibits the growth of metastatic breast and prostate tumors, but mechanical stimulation may reverse this effect. Moreover, it demonstrated that mechanically stimulating bone cells increases the invasion of breast and prostate cancer cells.

## 5. Bone Organoid-on-a-Chip

### 5.1. Construction of Bone Organoids

Bone organoids are miniature 3D self-renewing and self-organizing bone tissues. They are constructed based on bioactive materials and formed through the directed differentiation of stem cells (such as MSCs, embryonic stem cells, etc.) or progenitor cells (like osteoblasts, osteoclasts, etc.) [71]. These organoids possess biomimetic three-dimensional spatial characteristics, allowing them to simulate the structural and complex physiological functions of bone under physiological conditions [72]. Therefore, bone organoids provide a powerful model for studying bone development, the dynamic regulation of bone, and tissue regeneration. Moreover, they have opened up new avenues for drug screening and disease diagnostics.

**Figure 5:**
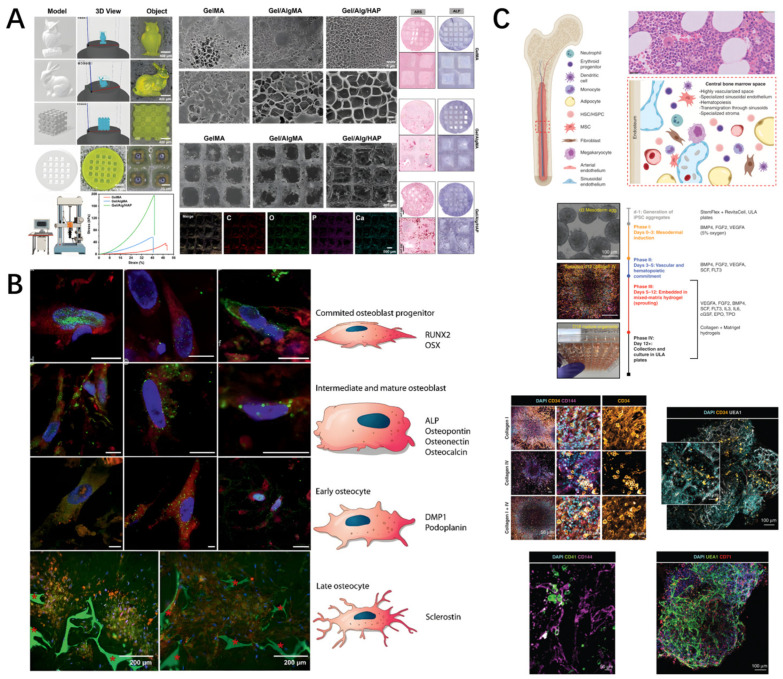
The construction of bone organoids. (**A**) Large-scale 3D bone organoids loaded with BMSCs bioprinted using GelMA/AlgMA/HAP bioink (composition: 5% GelMA, 1% AlgMA, 10% HAP nanoparticles); organoids cultured for 28 days show progressive mineralization (alizarin red staining intensity increases over time); in vivo implantation in a rat calvarial defect model demonstrates new bone formation (blue staining) and vascular ingrowth (CD31-positive vessels) at 8 weeks post-implantation. Reprinted with permission from Ref. [73]. (**B**) Stepwise differentiation of hBMSCs into osteoblasts and osteocytes within woven bone organoids; immunofluorescence shows osteoblast marker RUNX2 (day 7), osteocyte markers DMP1 and sclerostin (day 21), and dendritic osteocyte processes forming cellular networks embedded in self-produced mineralized collagen matrix (day 28); scale bar: 10 μm. Reprinted with permission from Ref. [74]. (**C**) Vascularized bone marrow organoids constructed by regulating iPSC proliferation and differentiation in Matrigel/collagen mixed matrix; CD34+ hematopoietic progenitors and CD31+ endothelial cells self-organize into vascular networks supporting hematopoiesis; organoids show bone marrow cavity-like structures with osteogenic (RUNX2+), endothelial (CD31+), and hematopoietic (CD45+) compartments after 21 days of culture. Reprinted with permission from Ref. [75].

J Su et al. [73] have developed a novel bio-ink composed of methacrylated gelatin/methacrylated alginate/hydroxyapatite (GelMA/AlgMA/HAP). By mimicking the natural bone ECM, this bio-ink is used for large-scale 3D bioprinting of bone organoids, as shown in Figure 5A. Both in vitro and in vivo experiments have demonstrated that it can significantly promote bone tissue formation and bone defect repair, offering a new strategy for regenerative medicine.

Woven bone organoids are a structural form of bone tissue that is embryonic and involved in fracture repair. Their main characteristic is the irregular interweaving arrangement of collagen fibers, which are subsequently gradually remodeled into lamellar bone [72]. In a study, researchers developed an organoid for woven bone by directing the differentiation of human bone marrow stromal cells into osteoblasts and osteocytes, achieving three-dimensional self-renewal and co-culture, thereby creating an organoid for the formation of early bone (woven bone), as shown in Figure 5B [74].

The bone marrow microenvironment, composed of a multitude of cells, maintains hematopoietic function [66]. In a study, researchers successfully constructed a three-dimensional structure that mimics the vascularized human bone marrow cavity by regulating the proliferation and differentiation of induced pluripotent stem cells (iPSCs) within a mixed matrix hydrogel containing Matrigel and type I and type IV collagens, as shown in Figure 5C [75].

Callus organoids are newly formed osteoid tissue produced during fracture healing or long-bone development and can persist in subsequent osteogenesis. In a study, researchers used GelMA hydrogel microspheres and digital light processing (DLP) 3D printing technology to successfully prepare microspheres containing bone MSCs, These were then induced to form callus organoids in a chondrogenic medium in vitro [76].

Chondrogenic organoids can be generated by differentiating iPSCs towards the mesodermal lineage, followed by self-organization without scaffolds, and further differentiation into chondrocytes. These cells then self-assemble into chondrogenic organoids [77].

Trabecular bone is one of the main forms of skeletal structure. In the process of preparing trabecular bone organoids, researchers first fabricate demineralized bone paper (DBP). They then culture osteoblasts on the DBP and induce their differentiation into bone lining cells. Subsequently, they simulate the bone remodeling cycle by co-culturing osteoblasts with bone marrow mononuclear cells. Finally, they construct organoid models using DBP discs of varying sizes and spacers of different heights to mimic the absorption and remodeling of trabecular bone under physiological conditions [78].

### 5.2. The Application of Bone Organoids

In recent years, significant progress has been made in the research of bone organoids. By integrating 3D printing technology and hydrogel materials and further inducing them, researchers have achieved long-term culture and maturation of bone organoids. Additionally, by regulating the proliferation and differentiation of iPSCs, specific bone organoids can be generated. These bone organoids can be used to develop disease models for bone disorders. Compared with traditional 2D cultures that lack three-dimensional structures, bone organoids contain multiple cell types and ECM components, better simulating the complexity of bone tissue. Compared with animal models, bone organoids can eliminate interspecies differences and more accurately simulate the human pathological microenvironment, thereby facilitating the study of disease mechanisms [79].In specific research, for instance, in the study of osteoporosis, specific cytokines that regulate and promote or inhibit bone formation can be introduced into bone organoid models to accelerate bone resorption relative to bone formation, thereby establishing disease models [72]. In the study of breast cancer bone metastasis, researchers cultured MSCs on a 3D bone scaffold model and induced their differentiation into osteoblasts, thereby creating a microenvironment that mimics trabecular bone. Subsequently, breast cancer cells were introduced into this model to construct a three-dimensional model of breast cancer bone metastasis [80]. Moreover, organoid models can be used to evaluate drugs for the clinical treatment of bone diseases, thereby reducing the duration of the drug-testing cycle. Additionally, these models can be used to assess the biocompatibility of implanted biomaterials by constructing in vitro bone organoids, thereby reducing reliance on animal experimentation. In the field of bone repair and regeneration, bone-related cells and osteogenic growth factors produced by bone organoids can serve as bioactive components to promote bone regeneration or enhance the regenerative and reparative capabilities of conventional tissue-engineered biomaterials [81].

### 5.3. Bone Organoids Cultured on a Chip

However, bone organoids may still face challenges, including insufficient vascularization, limited nutrient supply, and the absence of mechanical stimulation. These issues can be addressed by integrating bone organoids with microfluidic organ-on-a-chip technology, which enables precise control of fluid flow to provide a dynamic culture environment, simulate blood flow, construct vascularized models, and apply mechanical forces to mimic biomechanical conditions. This integration can be utilized for disease modeling, drug screening, personalized medicine, and toxicity testing. Future research directions may include maintaining long-term stable culture, integrating vascular networks into vascularized bone organoids, simulating complex mechanical environments such as the various mechanical stimuli experienced by bones, and achieving high-throughput drug screening. Additionally, by using microfluidic chips for multi-organ integration, multi- organ-on-a-chips can be constructed. By implanting bone organoids into microfluidic chips, the biomechanical relationships between three-dimensional organoids can be better simulated and studied. The development of this technology provides new perspectives and tools for the research and treatment of bone diseases.

## 6. Summary and Outlook

Bone-on-a-chip technology has achieved significant progress in simulating the bone tissue microenvironment, investigating bone remodeling mechanisms, and modeling bone-related diseases. Current models have enabled various functions including osteoblast-osteoclast co-culture, vascularized bone tissue construction, bone marrow hematopoietic niche simulation, and bone metastasis tumor research. However, existing models still exhibit notable limitations in replicating the physiological complexity of bone tissue, including limited cell types, simplified bone matrix structures, monotonic mechanical stimulation patterns, and a lack of functional vasculature and innervation. These bottlenecks constrain the predictive capability and clinical translation potential of the models.

The future development of bone-on-a-chip must first achieve breakthroughs at the material level. Current models predominantly employ single bone-mimicking materials (such as HA, β-TCP, or polyHIPE), whereas native bone tissue exhibits spatial heterogeneity between cortical and cancellous bone as well as hierarchical pore size distributions (ranging from submicron canaliculi to hundred-micron Haversian canals). Developing gradient pore scaffolds and composite scaffold materials can more accurately reproduce the hierarchical architecture of the bone matrix. Concurrently, constructing dynamic mineralized matrices to simulate the progressive mineralization during bone formation will provide cells with growth environments closer to physiological conditions.

Regarding cellular complexity, current bone-on-a-chip models primarily focus on three-way interactions among osteoblasts, osteoclasts, and osteocytes. However, the bone tissue microenvironment also comprises multiple components including immune cells (such as macrophages and T cells), mesenchymal stromal cells, adipocytes, and vascular endothelial cells. Macrophages, in particular, possess dual roles in promoting or suppressing inflammation during bone remodeling and can regulate the balance between bone formation and resorption—a mechanism that has not been adequately investigated in chip models. Integrating these immune cells will significantly enhance the predictive capacity of models for inflammatory bone diseases (such as rheumatoid arthritis) and bone metastasis research.

Vascularization and innervation represent critical directions for bone-on-a-chip functionalization. Existing vascularized bone chips predominantly utilize co-cultures of vascular endothelial cells and mesenchymal stem cells but lack perfusable vascular systems with authentic blood flow functionality. Future efforts need to construct perfusable three-dimensional vascular networks to simulate shear forces generated by blood flow within bone and nutrient/oxygen gradients. Furthermore, bone tissue is under dual regulation by sensory and sympathetic nerves; neuropeptides (such as CGRP) can influence osteoblast function and play important roles in pain perception and bone metabolism. The integration of innervation will expand the application of bone-on-a-chip in studying bone pain mechanisms.

From a systemic perspective, bone metabolism is regulated by systemic hormones (such as parathyroid hormone and estrogen) and inter-organ interactions (such as gut-bone and kidney-bone axes). Integrating bone-on-a-chip with liver-on-a-chip, kidney-on-a-chip, and intestine-on-a-chip to construct multi-organ combined systems can be used to study systemic bone metabolic diseases and drug metabolism processes in vivo, holding significant value for evaluating systemic side effects of anti-resorptive drugs and renal clearance mechanisms.

The integration of bone organoids with chip technology represents another important trend. Bone organoids possess the potential for self-organization into bone tissue but face challenges of insufficient vascularization and poor long-term culture stability. Implanting bone organoids into microfluidic chips and precisely controlling fluid flow, nutrient supply, and mechanical stimulation can promote organoid maturation. Future efforts need to develop standardized bone organoid culture protocols, achieve self-assembly of vascular networks within organoids, and establish patient-derived bone organoid biobanks for personalized drug screening.

The translation of bone-on-a-chip from laboratory to clinic also requires addressing standardization and reproducibility issues. Current models lack unified standards in chip design, material preparation, cell sources, and culture conditions, making cross-platform comparison of experimental results difficult. Establishing standard operating procedures, developing modular chip components, and automated culture systems are prerequisites for achieving high-throughput drug screening and safety evaluation. Additionally, patient-derived stem cell-based bone-on-a-chip models will provide personalized drug efficacy prediction platforms for precision medicine, though issues of cell differentiation variability and long-term culture costs need to be resolved.

Finally, the integration of real-time monitoring technologies will enhance the analytical capabilities of bone-on-a-chip. Existing models predominantly rely on endpoint detection (such as histological staining and genetic analysis) and lack real-time observation capabilities for dynamic bone remodeling processes (such as mineralized deposition and bone resorption lacunae formation). Integrating multiple detection modalities including electrode sensing, microfluidic sampling, and optical imaging can construct multi-parameter monitoring platforms, enabling continuous observation and mechanistic analysis of bone remodeling processes.

In summary, bone-on-a-chip technology is advancing from proof-of-concept toward functionalization, complexity, and clinical translation. Through material innovation, enhanced cellular complexity, vascular and neural integration, and multi-organ system construction, future bone-on-a-chip systems are expected to become in vitro bone models more closely approximating human physiology, providing reliable technological platforms for bone disease mechanism research, drug development, and regenerative medicine.

## Data Availability

No new data were created or analyzed in this study.
